# Roles of peritoneal clearance and residual kidney removal in control of uric acid in patients on peritoneal dialysis

**DOI:** 10.1186/s12882-020-01800-1

**Published:** 2020-04-25

**Authors:** Xi Xiao, Hongjian Ye, Chunyan Yi, Jianxiong Lin, Yuan Peng, Xuan Huang, Meiju Wu, Haishan Wu, Haiping Mao, Xueqing Yu, Xiao Yang

**Affiliations:** 1 Department of Nephrology, The First Affiliated Hospital, Sun Yat-sen University, 58th, Zhongshan Road II, Guangzhou, 510080 China; 2 Key Laboratory of Nephrology, Committee of Health and Guangdong Province, Guangzhou, 510080 China

**Keywords:** Peritoneal dialysis, Uric acid, Clearance, Residual kidney function

## Abstract

**Background:**

There have been few systematic studies regarding clearance of uric acid (UA) in patients undergoing peritoneal dialysis (PD). This study investigated peritoneal UA removal and its influencing factors in patients undergoing PD.

**Methods:**

This cross-sectional study enrolled patients who underwent peritoneal equilibration test and assessment of Kt/V from April 1, 2018 to August 31, 2019. Demographic data and clinical and laboratory parameters were collected, including UA levels in dialysate, blood, and urine.

**Results:**

In total, 180 prevalent patients undergoing PD (52.8% men) were included. Compared with the normal serum UA (SUA) group, the hyperuricemia group showed significantly lower peritoneal UA clearance (39.1 ± 6.2 vs. 42.0 ± 8.0 L/week/1.73m^2^; *P* = 0.008). Furthermore, higher transporters (high or high-average) exhibited greater peritoneal UA clearance, compared with lower transporters (low or low-average) (42.0 ± 7.0 vs. 36.4 ± 5.6 L/week/1.73 m^2^; *P* < 0.001). Among widely used solute removal indicators, peritoneal creatinine clearance showed the best performance for prediction of higher peritoneal UA clearance in receiver operating characteristic curve analysis [area under curve (AUC) 0.96; 95% confidence interval [CI], 0.93–0.99]. Peritoneal UA clearance was independently associated with continuous SUA [standardized coefficient (β), − 0.32; 95% CI, − 6.42 to − 0.75] and hyperuricemia [odds ratio (OR), 0.86; 95% CI, 0.76–0.98] status, only in patients with lower (≤2.74 mL/min/1.73 m^2^) measured glomerular filtration rate (mGFR). In those patients with lower mGFR, lower albumin level (β − 0.24; 95%CI − 7.26 to − 0.99), lower body mass index (β − 0.29; 95%CI − 0.98 to − 0.24), higher transporter status (β 0.24; 95%CI 0.72–5.88) and greater dialysis dose (β 0.24; 95%CI 0.26–3.12) were independently associated with continuous peritoneal UA clearance. Furthermore, each 1 kg/m^2^ decrease in body mass index (OR 0.79; 95% CI 0.63–0.99), each 1 g/dL decrease in albumin level (OR 0.08; 95%CI 0.01–0.47), and each 0.1% increase in average glucose concentration in dialysate (OR 1.56; 95%CI 1.11–2.19) were associated with greater peritoneal UA clearance (> 39.8 L/week/1.73m^2^).

**Conclusions:**

For patients undergoing PD who exhibited worse residual kidney function, peritoneal clearance dominated in SUA balance. Increasing dialysis dose or average glucose concentration may aid in controlling hyperuricemia in lower transporters.

## Background

Uric acid (UA, 2,6,8-trihydroxypurine; C5H4N4O3), as the end-products of endogenous and dietary purine metabolism, is a weak diprotic acid that possesses two dissociable protons with a pKa1 of 5.4 and pKa2 of 10.3, respectively [1]. At a physiology PH of 7.4, 98% of UA exists as monosodium urate in the extracellular milieu [2]. UA is poorly soluble in aqueous media and cannot freely move through the cytomembrane; therefore, it is excreted mainly by means of UA transporters, generally located in the kidney and intestines. Reportedly, approximately 70% of UA is excreted by the kidney, while 30% is excreted by the gastrointestinal tract [3, 4]. Because of the important role of the kidney in excreting UA and maintaining UA balance in the internal environment, nearly 90% hyperuricemia is caused by impairment of renal UA excretion [5]. Similarly, hyperuricemia is common in patients with chronic kidney disease; these patients exhibit fivefold greater prevalence of hyperuricemia than patients with normal renal function [6].

Peritoneal dialysis (PD), a widely used dialysis modality, is becoming increasingly important in renal replacement therapy for patients with end-stage renal disease for its cost-effectiveness and related improvements in techniques and patient survival [7]. The prevalence of hyperuricemia increases with decline in renal function, these prevalences range from 40 to 70% in patients with chronic kidney disease stages 1–5 [8–10]. In patients receiving dialysis, the prevalence reportedly increased with increasing dialysis vintage, and are similar in patients undergoing hemodialysis and those undergoing PD [9, 11]. The effect of SUA on prognosis among patients undergoing dialysis is controversial. Most of studies of patients undergoing hemodialysis showed that the lower SUA level was a risk factor for mortality [12–14]. However, the higher SUA level was shown to be independently associated with mortality in patients undergoing PD [15–17], though some studies revealed no association [13, 18]; notably, one study recently showed an inverse association [19]. These inconsistent results between hemodialysis and PD therapies were reportedly partially related to the kinetics of UA clearance in each dialysis regimen [2]. To the best of our knowledge, there have been relatively few studies regarding UA clearance, especially in patients undergoing PD. In addition to the effects of UA-lowering agents and optimizing dietary and lifestyle factors [20], dialysis therapy itself plays a role in SUA control in patients undergoing PD [21]. However, the relative role of peritoneal UA clearance and residual renal removal in achievement of adequate SUA homeostasis have not been studied. Here, we systematically investigated the contributions of peritoneal UA clearance with respect to residual kidney function and identified its relevant modifiable factors of dialysis prescription in patients undergoing PD.

## Methods

### Study population

This single-center cross-sectional study enrolled patients who had undergone peritoneal equilibration test (PET) and assessment of Kt/V in our PD center from April 1, 2018 to August 31, 2019. The inclusion criteria included prevalent patients aged ≥18 years who had initiated PD therapy at least 1 month prior to PET and Kt/V tests. Patients were excluded if they had taken UA-lowering agents within 1 month before PET and Kt/V tests, had transferred from long-term hemodialysis (i.e., longer than 3 months), had undergone failed renal transplantation, or exhibited malignant tumors. All enrolled patients used standard lactate-glucose peritoneal dialysate (1.5, 2.5%, or 4.25% dextrose; Baxter, Guangzhou, China). Relevant clinical parameters were tested in the clinical laboratory of the First Affiliated Hospital of Sun Yat-sen University using standard methods. All patients provided written informed consent to participate. The study was performed in accordance with the ethical principles of the Declaration of Helsinki and was approved by the Clinical Research Ethics Committee of the First Affiliated Hospital of Sun Yat-sen University.

### Data collection

Demographic data were collected, included age, sex, body mass index (BMI), diabetes status, cardiovascular disease status, and primary kidney disease. Data of the first PET and Kt/V tests during the study period were collected. The PD-related data that were collected included dialysis vintage, dialysis dose, average glucose concentration in dialysate, measured glomerular filtration rate (mGFR), Kt/V, weekly total creatinine clearance (CCL), 24 h residual urine volume, normalized protein catabolic rate and standard PET data. The standard PET data described the urea, creatinine, and UA levels in dialysate, blood, and urine samples with 2 L of 2.5% dextrose dialysate dwelling for 0, 2 or 4 h; 0 h was the time point in the PET test when all of the 2 L dialysate flowed into abdominal cavity, and the duration of this process was recorded. Patients undergoing PD were classified into high, high average, low average, or low transporters, in accordance with Twardowski’s criterion [22]. Clinical parameters included blood pressure, hemoglobin, neutrophil/lymphocyte ratio, high sensitivity C-reactive protein, serum albumin, prealbumin, corrected calcium, phosphorus, total cholesterol, triglyceride, serum urea nitrogen, creatinine, SUA and intact parathyroid hormone. Medication history was also collected, using follow-up records of patients who regularly visited our PD center for assessment and therapeutic regimen adjustment at 1–3-month intervals. Cardiovascular disease was defined as current or prior angina, myocardial infarction, congestive heart failure, cerebrovascular events, or peripheral vascular disease [23]. The charlson comorbidity score was used to evaluate the comorbidities of enrolled patients [24]. Men with SUA > 420 μmol/L or women with SUA > 360 μmol/L were regarded as hyperuricemic. The data of mGFR, Kt/V, CCL and normalized protein catabolic rate were obtained using PD Adequest software 2.0 (Baxter, Deerfield, IL, USA). Body surface area (BSA) was calculated using the well-known DuBois & DuBois formula [25]. UA clearance was calculated using the following formulae:

$$ \operatorname{Re}\mathrm{nal}\;\mathrm{UA}\;\mathrm{clearance}\left(\mathrm{L}/\mathrm{week}/1.73{\mathrm{m}}^2\right)=\frac{{\mathrm{UA}}_{\mathrm{urine}}\left(\upmu \mathrm{mol}/\mathrm{L}\right)\times 24\mathrm{h}\;\mathrm{urine}\kern0.17em \mathrm{output}\left(\mathrm{L}\right)\times 7\times 1.73\left({m}^2\right)}{\mathrm{SUA}\left(\upmu \mathrm{mol}/\mathrm{L}\right)\times \mathrm{BSA}\left({m}^2\right)} $$

$$ \mathrm{Peritoneal}\kern0.5em \mathrm{UA}\ \mathrm{clearance}\left(\mathrm{L}/\mathrm{week}/1.73{\mathrm{m}}^2\right)=\frac{{\mathrm{UA}}_{\mathrm{dialysate}}\left(\upmu \mathrm{mol}/\mathrm{L}\right)\times 24\mathrm{h}\;\mathrm{dialysate}\kern0.17em \mathrm{output}\left(\mathrm{L}\right)\times 7\times 1.73\left({m}^2\right)}{\mathrm{SUA}\left(\upmu \mathrm{mol}/\mathrm{L}\right)\times \mathrm{BSA}\left({m}^2\right)} $$

$$ \mathrm{Total}\ \mathrm{UA}\ \mathrm{clearance}=\mathrm{Renal}\ \mathrm{UA}\ \mathrm{clearance}+\mathrm{Peritoneal}\ \mathrm{UA}\ \mathrm{clearance} $$

### Statistical analysis

Enrolled patients were divided into two groups according to median peritoneal UA clearance. Data are presented as mean ± standard deviation for normally distributed continuous variables, medians (interquartile range) for non-normally distributed continuous variables, and frequencies and percentages for categorical variables. Differences between the lower and higher peritoneal UA clearance groups were analyzed using independent samples t-tests for normally distributed continuous variables, the Mann–Whitney U test for non-normally distributed continuous variables, and chi-squared tests for categorical variables. Pearson correlation or Spearman rank correlation test were used to evaluate correlations between variables of normal or skewed distribution, respectively. Multiple linear regression and binary logistic regression were performed to explore the independent influencing factors of continuous and categorical SUA and peritoneal UA clearance in total, lower (≤2.74 mL/min/1.73m^2^) and higher (> 2.74 mL/min/1.73m^2^) mGFR group, respectively. Following exclusion of the potential effects of multicollinearity, variables that were significant in univariate analysis (*P* < 0.05) and those that exhibited clinical correlations were entered into the final model. The performances of small solute removal indicators for prediction of higher peritoneal UA clearance were tested using area under the receiver operating characteristic curve analysis. Two-sided *P* values < 0.05 were regarded as statistically significant. All statistical analyses were conducted in SPSS Statistics software (version 20.0; IBM Corp., Armonk, NY, USA).

## Results

### Patient characteristics

**Fig. 1:**
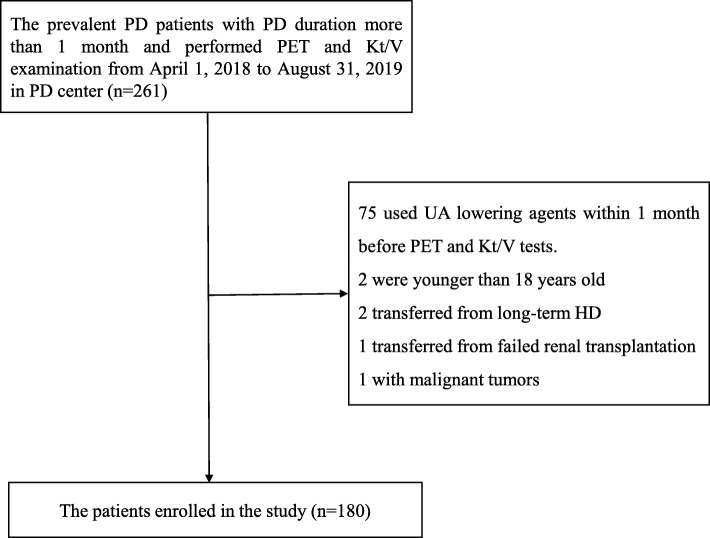
The flow chart for enrollment process of patients undergoing PD in the study. HD, hemodialysis; PD, peritoneal dialysis; PET, peritoneal equilibration test; UA, uric acid

As shown in Fig. 1, 180 patients were included in this study (mean age, 45.0 ± 13.4 years; 52.8% men; 13.3% with diabetes). Primary kidney diseases included chronic glomerulonephritis (67.2%), diabetic nephropathy (8.9%), hypertensive lesions (7.8%), and others (16.1%). The mean SUA level was 410 ± 72 μmol/L; 15.0% of patients used diuretics within 1 month before PET and Kt/V tests performed at enrollment. Patients with higher peritoneal UA clearance were older, had a greater proportion of women, and lower level of BMI, serum albumin, and SUA (Table 1). PD-related data are shown in Table 2. Overall, the patients had a median dialysis vintage of 1.6(1.4–19.8) months and a mean peritoneal UA clearance of 40.2 ± 7.1 L/week/1.73m^2^. Patients with higher peritoneal UA clearance had longer PD vintage, as well as higher average glucose concentration in dialysate, dialysis dose, total Kt/V, peritoneal Kt/V, and peritoneal CCL; they also had lower residual renal Kt/V, renal CCL, residual urine volume, and mGFR. Notably, there was a larger proportion of high transporters and a smaller proportion of low average transporters.

**Table 1:** The demographic characteristics of enrolled patients in the study

Variables	Total (*n* = 180)	Lower peritoneal UA clearance (*n* = 90)	Higher peritoneal UA clearance (*n* = 90)	*P* value
Peritoneal UA clearance(L/week/1.73 m^2^)	40.2 ± 7.1	34.6 ± 3.5	45.8 ± 5.0	*–*
Age (y)	45.0 ± 13.4	42.9 ± 13.2	47.1 ± 13.3	0.04
Male (n, %)	95 (52.8)	55 (61.1)	40 (44.4)	0.04
Diabetes (n, %)	24 (13.3)	13 (14.4)	11 (12.2)	0.83
CVD (n, %)	25 (13.9)	10 (11.1)	15 (16.7)	0.39
Charlson comorbidity score	3 (2–4)	2 (2–4)	3 (2–4)	0.09
Chronic glomerulonephritis (n, %)	121 (67.2)	63 (70.0)	58 (64.4)	0.53
Diabetic nephropathy (n, %)	16 (8.9)	8 (8.9)	8 (8.9)	1.00
Hypertensive kidney lesion (n, %)	14 (7.8)	5 (5.6)	9 (10.0)	0.41
Systolic pressure (mmHg)	135.0 ± 18.0	134.6 ± 17.4	135.5 ± 18.7	0.73
Diastolic pressure (mmHg)	85.5 ± 14.2	87.5 ± 12.7	83.5 ± 15.4	0.06
Hemoglobin (g/dL)	11.2 ± 1.7	11.4 ± 1.6	11.0 ± 1.7	0.11
N/L	3.5 (2.7–4.5)	3.2 (2.6–4.2)	3.7 (2.9–4.8)	0.11
HsCRP (mg/L)	1.4 (0.5–4.7)	1.5 (0.6–4.9)	1.3 (0.5–4.8)	0.79
Serum albumin (g/dL)	3.7 ± 0.4	3.8 ± 0.3	3.5 ± 0.4	<0.001
Serum prealbumin (mg/L)	351 (320–402)	354 (319–406)	347 (319–387)	0.26
Corrected calcium (mg/dL)	9.2 ± 1.0	9.1 ± 0.6	9.3 ± 1.2	0.22
Serum phosphorus (mg/dL)	4.5 ± 1.3	4.5 ± 1.1	4.5 ± 1.5	0.93
Total cholesterol (mg/dL)	193.4 (162.4–228.2)	193.4 (158.5–230.1)	193.4 (166.3–228.2)	0.62
Triglyceride (mg/dL)	136.4 (97.8–190.8)	142.1 (102.7–196.8)	131.9 (94.7–185.1)	0.27
Serum urea nitrogen (mg/dL)	45.5 (37.5–55.7)	45.4 (37.7–55.8)	45.9 (36.6–55.9)	0.91
Serum creatinine (mg/dL)	8.6 (7.3–10.8)	8.4 (7.1–10.8)	8.9 (7.3–10.9)	0.75
iPTH (pg/mL)	256.2 (149.4–401.4)	256.2 (164.0–391.1)	258.0 (139.6–454.3)	0.82
Serum UA (μmol/L)	410 ± 72	430 ± 71	391 ± 69	< 0.001
BMI (kg/m^2^)	21.8 ± 3.2	22.6 ± 3.2	21.0 ± 3.0	0.001
Diuretic use (n, %)	27 (15.0)	11 (12.2)	16 (17.8)	0.40

Values are presented as means ± standard deviation or medians (interquartile range) for continuous variables and count (percentage) for categorical variables. Enrolled patients were divided into lower peritoneal UA clearance (≤39.8 L/week/1.73m^2^) and higher peritoneal UA clearance (> 39.8 L/week/1.73m^2^) group according to its median level

*Abbreviations*: *BMI* body mass index, *CVD* cardiovascular disease, *HsCRP* high sensitivity C-reactive protein, *iPTH* intact parathyroid hormone, *N/L* neutrophil to lymphocyte ratio, *UA*, uric acid

**Table 2:** The PD-related information of patients

Variables	Total (*n* = 180)	Lower peritoneal UA clearance (*n* = 90)	Higher peritoneal UA clearance (*n* = 90)	*P* value
Peritoneal UA clearance(L/week/1.73 m^2^)	40.2 ± 7.1	34.6 ± 3.5	45.8 ± 5.0	*–*
PD vintage (m)	1.6 (1.4–19.8)	1.5 (1.3–8.4)	2.3 (1.4–49.9)	0.01
Average dialysate glucose concentration (%)	1.5 (1.5–1.7)	1.5 (1.5–1.5)	1.5 (1.5–1.8)	< 0.001
Dialysis dose (L/d)	8.103 ± 0.843	7.939 ± 0.800	8.267 ± 0.859	0.006
DAPD (n, %)	12 (6.7)	4 (3.2)	8 (4.8)	0.37
Total Kt/V	2.3 ± 0.5	2.2 ± 0.4	2.4 ± 0.6	0.03
Residual renal Kt/V	0.7 ± 0.5	0.8 ± 0.4	0.6 ± 0.5	0.02
Peritoneal Kt/V	1.7 ± 0.3	1.5 ± 0.3	1.8 ± 0.3	< 0.001
CCL (L/week/1.73m^2^)	76.1 (60.4–93.1)	76.8 (63.2–94.7)	74.7 (59.0–91.5)	0.36
Residual renal CCL (L/week/1.73m^2^)	30.4 (10.4–49.8)	38.3 (20.4–56.1)	22.5 (1.5–44.0)	< 0.001
Peritoneal CCL (L/week/1.73m^2^)	46.1 (41.0–51.3)	41.3 (38.7–43.2)	50.9 (48.0–56.6)	< 0.001
mGFR (mL/min/1.73m^2^)	3.1 ± 2.4	3.8 ± 2.4	2.3 ± 2.3	< 0.001
Residual urine volume (L)	0.700 (0.300–1.200)	0.850 (0.550–1.225)	0.555 (0.050–1.100)	0.003
nPCR (g/kg/d)	0.889 (0.769–1.076)	0.856 (0.765–1.028)	0.934 (0.785–1.126)	0.05
PET category (%)				
High	15 (8.3)	1 (1.1)	14 (15.6)	0.001
High average	107 (59.4)	47 (52.2)	60 (66.7)	0.07
Low average	54 (30.0)	38 (42.2)	16 (17.8)	0.001
Low	4 (2.2)	4 (4.4)	0 (0.0)	0.13

Values are presented as means ± standard deviation or medians (interquartile range) for continuous variables and count (percentage) for categorical variables. Enrolled patients were divided into lower peritoneal UA clearance (≤39.8 L/week/1.73m^2^) and higher peritoneal UA clearance (> 39.8 L/week/1.73m^2^) group according to its median level

*Abbreviations*: *CCL* creatinine clearance, *DAPD* Day Ambulatory Peritoneal Dialysis, *mGFR* measured glomerular filtration rate, *nPCR* normalized protein catabolic rate, *PD* peritoneal dialysis, *PET* peritoneal equilibration test, *UA* uric acid

### Relationships between peritoneal UA clearance and peritoneal transport characteristics

**Fig. 2:**
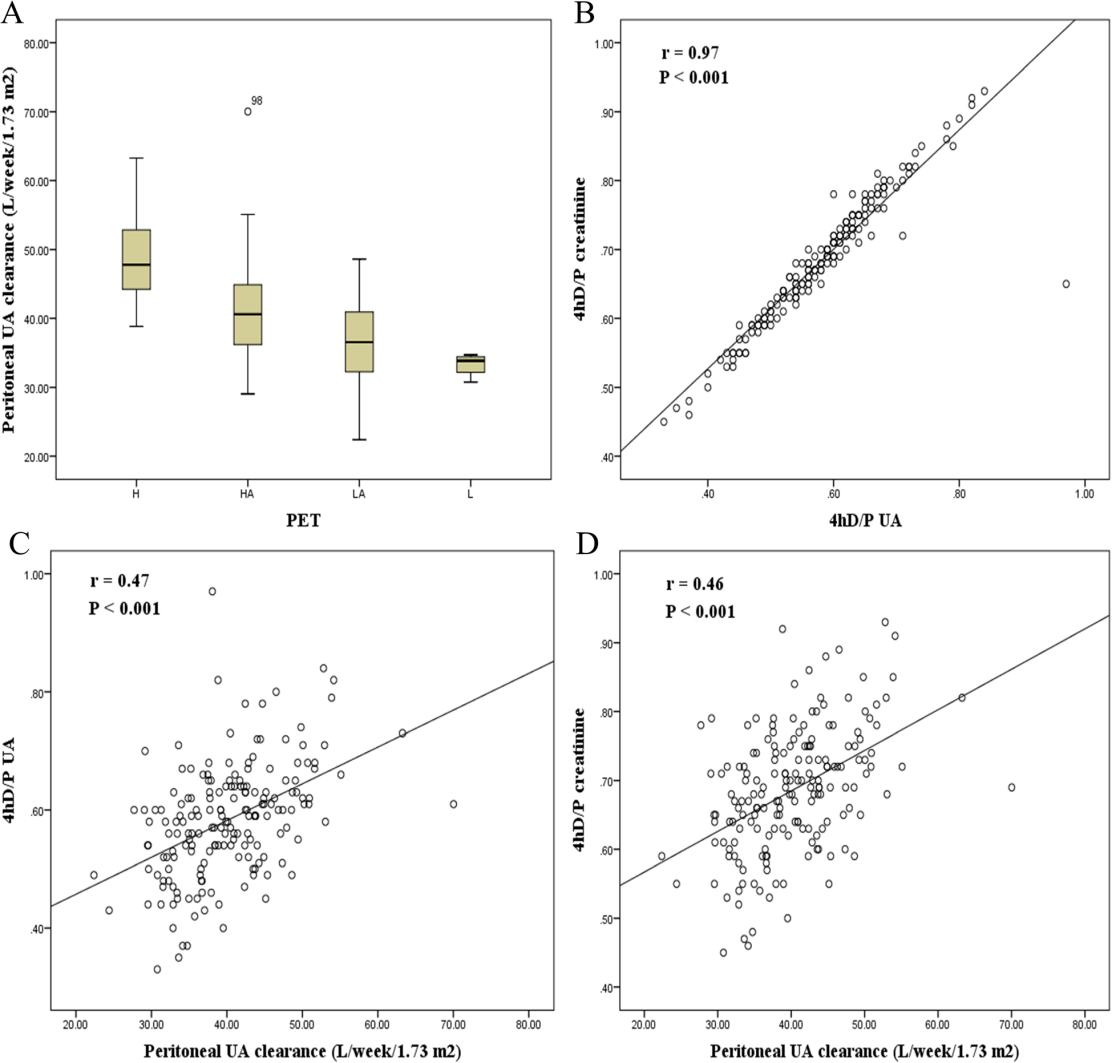
The effects of peritoneal transport characteristics on peritoneal UA clearance. **a** Distribution of peritoneal UA clearance according to peritoneal transport characteristics. **b** Correlation between the 4 h D/P creatinine and 4 h D/P UA. **c** Correlation between 4 h D/P UA and peritoneal UA clearance. **d** Correlation between 4 h D/P creatinine and peritoneal UA clearance. D/P, dialysate to plasm; UA, uric acid

**Fig. 3:**
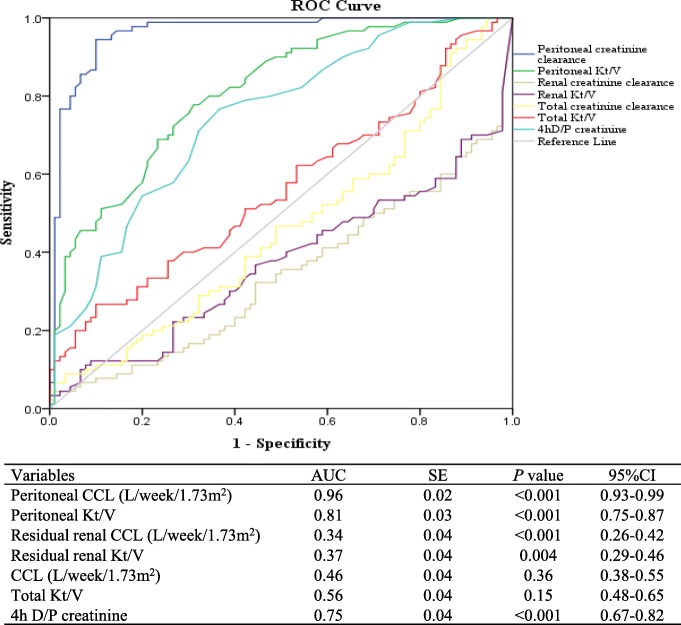
The performance of different small solute removal indicators for prediction of higher peritoneal UA clearance (> 39.8 L/week/1.73 m^2^) in receiver operating characteristic curve analysis. CCL, creatinine clearance; CI, confidence interval; D/P, dialysate to plasm; UA, uric acid

Distributions of peritoneal UA clearance according to peritoneal transport characteristics are shown in Fig. 2a; in particular, there was a progressive increase in peritoneal UA clearance with increasing peritoneal transport rate. Notably, higher transporters (high or high average) exhibited significantly greater peritoneal UA clearance, compared with lower transporters (low average or low) (42.0 ± 7.0 vs. 36.4 ± 5.6 L/week/1.73 m^2^; *P* < 0.001). As shown in Fig. 2b, 4h dialysate to plasma (D/P) UA was strongly correlated with 4 h D/P creatinine (*r* = 0.97; *P* < 0.001). Moreover, correlations were similar between 4 h D/P UA and peritoneal UA clearance (*r* = 0.47; *P* < 0.001) and between 4 h D/P creatinine and peritoneal UA clearance (*r* = 0.46; *P* < 0.001) (Fig. 2c and d). Among widely used small solute removal indicators, peritoneal CCL showed the best performance in receiver operating characteristic curve analysis [area under curve (AUC), 0.96; 95% confidence interval (CI), 0.93–0.99; *P* < 0.001] for prediction of higher peritoneal UA clearance (Fig. 3).

### Relationships of peritoneal UA removal with SUA in patients undergoing PD

**Fig. 4:**
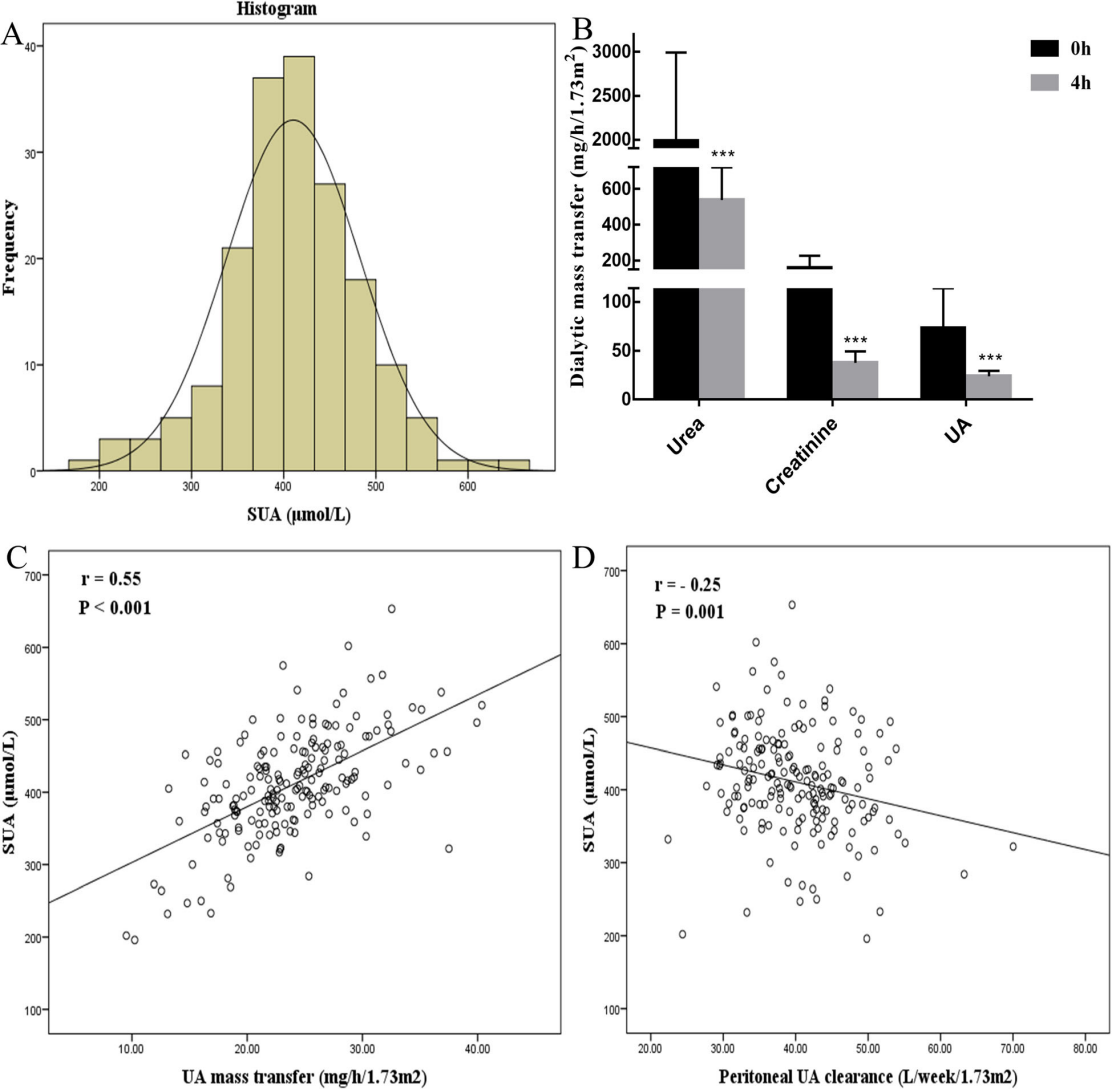
The correlation between peritoneal UA clearance and SUA. **a** Distribution of SUA in PD patients enrolled. **b** Average dialytic mass transfer of urea, creatinine and UA with 2 L of 2.5% glucose-based dialysate for dwell times of 0 and 4 h. **c** Correlation between the 4 h UA mass transfer and SUA. **d** Correlation between peritoneal UA clearance and SUA. ***P < 0.001, 0 h vs 4 h. SUA, serum uric acid; UA, uric acid

The distribution of SUA in patients undergoing PD is shown in Fig. 4a. The average mass transfers of urea, creatinine or UA with 2 L of 2.5% dextrose dialysate for dwell times of 0 and 4 h among all patients undergoing PD are described in Fig. 4b. Similar to the mass transfer of the small molecules of urea and creatinine, the peritoneal mass transfer of UA declined remarkably as dwell time increased. Whereas the average UA mass transfer for 4 h dwell time was positively correlated with SUA (*r* = 0.55; *P* < 0.001), peritoneal UA clearance was negatively correlated with SUA (*r* = − 0.25; *P* = 0.001) (Fig. 4c and d). In comparison with the normal SUA group, the hyperuricemia group showed significantly lower peritoneal UA clearance (39.1 ± 6.2 vs. 42.0 ± 8.0 L/week/1.73 m^2^; *P* = 0.008). The further analysis of multiple linear regression and binary logistic regression shown in Table 3 revealed that peritoneal UA clearance was independently associated with continuous SUA (β − 0.32; 95%CI − 6.42 to − 0.75; *P* = 0.01) and hyperuricemia status (OR 0.86; 95%CI 0.76–0.98; *P* = 0.02), only in patients undergoing PD who had lower mGFR.

**Table 3:** The relationships between peritoneal UA clearance and SUA in linear regression and logistic regression model in total, lower and higher mGFR patients, respectively

Variables	Continuous SUA		Hyperuricemia^a^	
	β (95%CI)	*P*^b^ value	Adjusted OR (95%CI)	*P*^b^ value
Total (*n* = 180)	−0.21(− 3.85, − 0.38)	0.02	0.91 (0.84, 0.98)	0.02
Lower mGFR group^c^ (*n* = 91)	−0.32(−6.42, − 0.75)	0.01	0.86 (0.76, 0.98)	0.02
Higher mGFR group (*n* = 89)	−0.10(− 3.55, 1.42)	0.40	0.94 (0.82, 1.06)	0.30

*Abbreviations*: *CI* confidence interval, *mGFR* measured glomerular filtration rate, *OR* odds ratio, *SUA* serum uric acid, *UA* uric acid

^a^Hyperuricemia was defined as men with SUA > 420 μmol/L or women with SUA > 360 μmol/L in the logistic regression model

^b^*P* value after adjusting the age, sex, diabetes, cardiovascular disease, dialysis vintage, mean arterial pressure, body mass index, albumin, normalized protein catabolic rate, mGFR, peritoneal UA clearance and use of diuretics in the multiple linear regression and the binary logistic regression models

^c^Did not adjust mGFR when analyzing in higher(> 2.74 mL/min/1.73m^2^) or lower (≤ 2.74 mL/min/1.73m^2^) mGFR group, respectively

### Independent factors influencing peritoneal UA clearance

As shown in Table 4, after adjusting for relevant demographic and PD-related variables in the multiple linear regression model, serum albumin level (β − 0.24; 95%CI − 7.26 to − 0.99; *P* = 0.01) and BMI (β − 0.29; 95%CI − 0.98 to − 0.24; *P* = 0.001) were both negatively associated with peritoneal UA clearance, while the higher transporter status (β 0.24; 95%CI 0.72–5.88; *P* = 0.01) and dialysis dose (β 0.24; 95%CI 0.26–3.12; *P* = 0.02) were positively associated with peritoneal UA clearance in the lower mGFR group. Similarly, binary logistic regression analysis revealed that each 0.1% increase in average glucose concentration in dialysate (OR 1.56; 95%CI 1.11–2.19; *P* = 0.01), each 1 g/dL decrease in albumin level (OR 0.08; 95%CI 0.01–0.47; *P* = 0.006), and each 1 kg/m^2^ decrease in BMI (OR 0.79; 95%CI 0.63–0.99; *P* = 0.04) were independently associated with greater peritoneal UA clearance (> 39.8 L/week/1.73m^2^) (Table 5).

**Table 4:** Associated factors of peritoneal UA clearance in multiple linear regression in total, lower and higher mGFR patients, respectively

Variables	Total		Lower mGFR group^a^		Higher mGFR group	
	β (95%CI)	*P* value	β (95%CI)	*P* value	β (95%CI)	*P* value
mGFR (mL/min/1.73m^2^)	− 0.08 (− 0.67, 0.18)	0.26	–	–	–	–
Age (y)	0.16 (0.02, 0.15)	0.01	0.10(− 0.03, 0.13)	0.23	0.24 (0.02, 0.26)	0.02
Sex (M/F)	−0.12 (− 3.35, 0.02)	0.053	− 0.14(− 3.97, 0.44)	0.12	−0.11(− 4.22,1.14)	0.26
Using diuretics	− 0.04 (− 3.04, 1.64)	0.56	− 0.007(− 3.04, 2.82)	0.94	−0.06(− 5.20,2.64)	0.52
Albumin (g/dL)	−0.15 (− 4.99, − 0.31)	0.03	−0.24(− 7.26, − 0.99)	0.01	−0.05(− 4.57,2.85)	0.64
PD vintage (month)	0.07 (−0.02, 0.05)	0.36	0.17(−0.006, 0.06)	0.10	−0.13(− 0.31,0.09)	0.28
BMI (kg/m^2^)	−0.27 (− 0.87, − 0.33)	<0.001	−0.29(− 0.98, − 0.24)	0.001	−0.32(− 1.10,-0.22)	0.004
Average glucose concentration of dialysate (0.1%)	0.14 (−0.005, 0.87)	0.05	0.19(−0.004, 0.88)	0.052	0.08(−0.70, 1.81)	0.38
Higher peritoneal transport status^b^	0.29 (2.40, 6.18)	<0.001	0.24 (0.72, 5.88)	0.01	0.32 (1.77,7.74)	0.002
Dialysis dose (L/d)	0.27 (1.10, 3.36)	<0.001	0.24 (0.26, 3.12)	0.02	0.20(−0.34,4.51)	0.09

*Abbreviations*: *BMI* body mass index, *CI* confidence interval, *mGFR* measured glomerular filtration rate, *PD* peritoneal dialysis, *UA* uric acid

^a^Did not adjust mGFR when analyzing in the higher(> 2.74 mL/min/1.73m^2^) or lower (≤ 2.74 mL/min/1.73m^2^) mGFR group, respectively

^b^The reference group was the lower (low average or low) peritoneal transporters

**Table 5:** Independent determinants of higher peritoneal UA clearance (> 39.8 L/week/1.73m^2^) in binary logistic regression in total, lower and higher mGFR patients, respectively

	Total		Lower mGFR group^a^		Higher mGFR group	
Variables	OR (95%CI)	*P* value	OR (95%CI)	*P* value	OR (95%CI)	*P* value
mGFR (mL/min/1.73m^2^)	0.96 (0.78, 1.18)	0.68	–	–	–	–
Age (y)	1.03 (1.00, 1.06)	0.07	1.01 (0.97, 1.05)	0.69	1.06 (1.01, 1.11)	0.03
Sex (M/F)	0.28 (0.12, 0.64)	0.003	0.28 (0.08, 1.00)	0.05	0.28 (0.09,0.90)	0.33
Using diuretics	0.63 (0.22, 1.85)	0.40	0.55 (0.11, 2.89)	0.48	0.81 (0.17,3.77)	0.78
Albumin (g/dL)	0.16 (0.05, 0.49)	0.001	0.08 (0.01, 0.47)	0.006	0.31 (0.06,1.54)	0.15
PD vintage (month)	1.00 (0.98, 1.02)	0.90	1.00 (0.98, 1.02)	0.97	0.99 (0.90,1.09)	0.82
BMI (kg/m^2^)	0.77 (0.67, 0.89)	0.001	0.79 (0.63, 0.99)	0.04	0.71 (0.57,0.89)	0.003
Average glucose concentration of dialysate (0.1%)	1.40 (1.07, 1.83)	0.02	1.56 (1.11, 2.19)	0.01	1.02 (0.58, 1.77)	0.96
Higher peritoneal transport status^b^	4.48 (1.73,11.56)	0.002	3.90 (0.97, 15.67)	0.06	5.65 (1.25,25.54)	0.02
Dialysis dose (L/d)	2.25 (1.23, 4.13)	0.009	2.16 (0.88, 5.35)	0.10	2.12 (0.76,5.91)	0.15

*Abbreviations*: *BMI* body mass index, *CI* confidence interval, *mGFR* measured glomerular filtration rate, *OR* odds ratio, *PD* peritoneal dialysis, *UA* uric acid

^a^Did not adjust mGFR when analyzing in the higher(> 2.74 mL/min/1.73m^2^) or lower (≤ 2.74 mL/min/1.73m^2^) mGFR group, respectively

^b^The reference group was the lower (low average or low) peritoneal transporters

## Discussion

The results of this study showed that peritoneal UA removal played a significant role in SUA control. Moreover, lower albumin and BMI, higher peritoneal transporter status, greater dialysis dose, and higher glucose concentration in dialysate were independently associated with greater peritoneal UA clearance in patients undergoing PD who had worse residual kidney function.

To the best of our knowledge, this is the first systematic analysis of UA clearance and factors that independently influenced UA clearance in patients undergoing PD. As a small molecular solute, the vast majority of UA is present in the ionized form; ≤5% of circulating UA is bound to albumin [4]. Because UA exhibits high hydrophilicity and has a sieving coefficient of 1.01, which allows it to easily diffuse through the dialysis membrane, it is presumed to be sufficiently cleared by PD therapy [2, 9]. A previous study showed that UA clearance was inversely proportional to the PD dwell time; specifically, the average UA mass transfer for dwell times of 0-1 h, 1-4 h and 4-8 h with 2 L of 1.5% dialysate were 49.8 ± 3.9, 16.1 ± 1.0, 8.3 ± 0.6 mg/h/1.73m^2^, respectively [26]. Similarly, we found remarkable reductions of UA mass transfer of 72.9 ± 41.1 and 23.9 ± 5.6 mg/h/1.73 m^2^ in PD with 2 L 2.5% dialysate for dwell times of 0 h and 4 h. In addition, we revealed an average peritoneal UA clearance of 40.2 ± 7.1 L/week/1.73m^2^ in patients undergoing PD.

In the present study, peritoneal UA clearance was significantly greater in higher transporters than in lower transporters, when measured in terms of 4 h D/P creatinine; moreover, 4 h D/P UA was strongly correlated with 4 h D/P creatinine. Further analysis revealed similar correlations between 4 h D/P UA and peritoneal UA clearance, as well as between 4 h D/P creatinine and peritoneal UA clearance. Moreover, receiver operating characteristic curve analysis revealed that, among widely used solute removal indicators, peritoneal CCL showed the best performance for prediction of higher peritoneal UA clearance. These results illustrated that membrane characteristics, assessed in terms of creatinine transport, can be used to determine UA transport status. This similarity is presumably because the molecular weight of UA (168 Da) is near that of creatinine (113 Da); in addition, few circulating UA molecules are bound to albumin or affected by electrochemical gradient, whereas serum phosphorus molecules are affected in this manner [27]. The present study revealed that evaluation of peritoneal UA clearance solely in terms of the most frequently used indicator for peritoneal adequacy (i.e., Kt/V) may not exhibit sufficient accuracy. Peritoneal CCL may be a more reliable index for assessing UA clearance adequacy. Adjustment of dialysis prescription for better PD-related UA removal, based on peritoneal CCL rather than the widely used Kt/V, is presumably more appropriate, particularly for lower transporters with hyperuricemia.

A negative correlation was observed between peritoneal UA clearance and SUA in the present study; further multiple linear and logistic regression analyses suggested that greater peritoneal UA clearance was significantly associated with lower SUA only in patients undergoing PD who had relatively low mGFR. This suggests that the kidney still plays an indispensable role in removing excessive SUA in patients with residual kidney function; and the importance of peritoneal UA clearance gradually became evident with the decline of residual kidney removal. Therefore, the high SUA in patients undergoing PD who had unsatisfying renal function may have been partially caused by inadequate UA removal during PD. In the present study, we found that lower BMI and albumin level, higher transporter status, greater dialysis dose, and higher glucose concentration in dialysate were significantly associated with greater peritoneal UA clearance in the lower mGFR group. BMI is a body composition parameter, which was strongly correlated with BSA in this study (data not shown); accordingly, patients undergoing PD who had lower BMI may exhibit greater peritoneal UA removal, after adjustment for their relatively lower BSA. In addition, the serum albumin level was revealed to be associated with both continuous peritoneal UA clearance and higher peritoneal UA clearance category in patients with worse renal function. Previous studies have shown negative correlations between peritoneal albumin loss and serum albumin level in patients undergoing PD [26, 28]. Furthermore, peritoneal albumin loss was demonstrated to be positively associated with peritoneal CCL in a cross-sectional study including 351 patients undergoing PD [29]. Therefore, a potential mechanism underlying the negative association between peritoneal UA clearance and serum albumin level is as follows: greater peritoneal UA clearance itself indicates greater removal of albumin from peritoneum, which causes lower circulating albumin reserves, primarily because of inadequate albumin synthesis to compensate peritoneal albumin loss [26]. Therefore, peritoneal albumin loss should be considered when optimizing dialysis prescription for efficient solute removal. However, it remains unclear whether there is a causal relationship between lower albumin level and greater peritoneal UA clearance.

Notably, few studies have explored PD-related factors associated with greater peritoneal UA clearance, particularly in terms of residual kidney function; average UA clearance in PD was revealed to be positively proportional to the exchange volume and flow rate [21]. In the present study, patients who were higher transporters exhibited greater peritoneal UA removal, compared with patients who were lower transporters. With the exception of non-modifiable peritoneal membrane characteristics, the modifiable dialysis dose factors significantly increase the PD-related UA removal as well. In patients with worse residual kidney function, average glucose concentration in dialysate tended to be associated with greater peritoneal UA removal, although this was not statistically significant (β 0.19; 95%CI − 0.004 to 0.88; *P* = 0.052); however, the effect was shown to be statistically significant in logistic regression (OR1.56; 95%CI 1.11–2.19; *P* = 0.01). Therefore, the positive effect of the glucose concentration in dialysate may have been masked by the relatively small sample size after grouping based on residual kidney function.

There were some limitations in our study. First, the cross-sectional observational study itself only assessed associations rather than causal relationships. Second, this study did not explore the effects of different PD modalities (e.g., continuous cyclic peritoneal dialysis, automated peritoneal dialysis) or different exchange flow rate on peritoneal UA clearance. Third, the dialysis vintages of enrolled patients were relatively short, which may have led to bias involving inadequate and unstable dialysis. Fourth, this study used a small sample size of patients without residual kidney function; therefore, classification of residual kidney function was grouped on the basis of median mGFR, rather than the clinical standards of oliguria or anuria. Despite the above limitations, to the best of our knowledge, this was the first study to systematically explore the contributions of peritoneal UA clearance and residual kidney removal, and to identify independent factors that influence peritoneal UA clearance. In this study, we concurrently collected common small solute removal indicators for further comparison and analysis, which provides important guidance in optimizing prescription for achievement of better SUA control in patients undergoing PD, especially those with worse residual kidney function. Moreover, we excluded patients undergoing PD who had a history of taking UA-lowering agents, which enabled us to more specifically study UA clearance in PD regimen.

## Conclusions

In summary, UA removal in patients undergoing PD was found to be more rely on peritoneal clearance, especially in patients with relatively worse residual kidney function. Peritoneal CCL may be an optimal indicator for assessment of UA removal during PD because of its similar removal characteristics through the dialysis membrane. For patients with unsatisfactory residual kidney function, increasing the dialysis dose or average glucose concentration in dialysate may aid in controlling hyperuricemia, particularly in patients who are lower transporters.

## Data Availability

The datasets used and analyzed during the current study are available from the corresponding author on reasonable request.

## Abbreviations

BMI: Body mass index
BSA: Body surface area
CCL: Creatinine clearance
CI: Confidence interval
D/P: Dialysate to plasm
mGFR: measured glomerular filtration rate
OR: Odds ratio
PD: Peritoneal dialysis
PET: Peritoneal equilibration test
SUA: Serum uric acid
UA: Uric acid
